# Cleft Sign in MRI May Represent the Disruption of Cartilage Structure within Pubic Symphysis and Pubic Plate: A Cadaver Case Report

**DOI:** 10.3390/diagnostics14182098

**Published:** 2024-09-23

**Authors:** Haruki Nishimura, Xueqin Gao, Sadao Niga, Naomasa Fukase, Yoichi Murata, Patrick M. Quinn, Masayoshi Saito, Hajime Utsunomiya, Soshi Uchida, Johnny Huard, Marc J. Philippon

**Affiliations:** 1 The Linda & Mitch Hart Center for Regenerative and Personalized Medicine, Steadman Philippon Research Institute, 181 West Meadow Dr., Suite 1000, Vail, CO 81657, USA; hnishimura2468@outlook.com (H.N.); xgao@sprivail.org (X.G.); pquinn0130@gmail.com (P.M.Q.); jhuard@sprivail.org (J.H.);; 2 Department of Orthopaedic Surgery and Sports Medicine, Wakamatsu Hospital of University of Occupational and Environmental Health, 1-17-1 Hamamachi, Wakamatsu, Kitakyushu-city 808-1264, Fukuoka, Japan; yoichi0928111@gmail.com (Y.M.); soushi@med.uoeh-u.ac.jp (S.U.); 3 JIN Orthopaedic & Sports Clinic, 3-10-7 Suzuya, Chuo-ku, Saitama-city 338-0013, Saitama, Japan; 4 Department of Orthopaedic Surgery, Kobe University, 7-5-2 Kusunoki-cho, Chuo-ku, Kobe-city 650-0017, Hyogo, Japan; nfukase@icloud.com; 5 Department of Orthopaedic Surgery, Hokusuikai Kinen Hospital, 3-2-1 Higashibaru, Mito-city 310-0035, Ibaraki, Japan; saitoma521@gmail.com; 6 Tokyo Sports & Orthopaedic Clinic, 4-29-9 Kamiikebukuro, Toyoshima-ku, Tokyo 170-0012, Japan; hajime.utsu@gmail.com; 7 The Steadman Clinic, 181 West Meadow Dr., Suite 1000, Vail, CO 81657, USA

**Keywords:** groin pain, cleft sign, pubic plate structure, human cadaver, cartilage

## Abstract

Background/Objectives: Long-standing groin pain is a severe issue for athletes, often associated with the cleft sign on magnetic resonance imaging (MRI) scans, yet its underlying causes are poorly understood. The purpose of this study is to histologically examine the pubic plate structure in cadavers with and without the cleft sign on MRI, shedding light on the pathology behind the cleft sign. Methods: Three fresh human pelvic cadavers underwent 3.0T MRI to detect the cleft sign before histological dissection of pubic plates. Pubic plate tissues were fixed in formalin, decalcified, and processed. Of the two cleft sign-negative specimens, one was cut into sagittal sections, and the other was cut into coronal sections for histology. For the cleft sign positive specimen, a sagittal section was cut. Moreover, 5 µm thick sections were cut at different axial levels for each orientation. Sections were subjected to Safranin O, Alcian blue, and Herovici’s staining or hematoxylin and eosin staining. Results: MRI confirmed that one specimen had a cleft sign in the inferior region on both sides of the pubis and that two specimens had no cleft sign. Both sagittal and coronal sections showed the presence of a cartilage structure continuing from the pubic symphysis to 3 mm laterally within the pubic plate. In the specimen with a positive cleft sign, cartilage damage within the pubic symphysis and pubic plate was identified as revealed by Safranin O staining, Herovici’s staining, and H&E staining. Conclusions: This study elucidated the existence of a cartilage component extending from the pubic symphysis to the pubic plate. The cleft sign in MRI correlated with a disruption in the cartilage component in histology within this specific area.

## 1. Introduction

Groin pain is typically defined as pain within the groin area, with additional classifications including groin disruption or athletic pubalgia, and frequently presents in athletes involved in sports that require sharp cutting movements [1,2]. In particular, it has been reported that the incidence of groin pain is greater and that the duration of return to sports is longer in soccer players than in other athletes [3,4]. According to the Doha Agreement, first formalized in 2015, groin pain is now recognized as a complex syndrome consisting of adductor-, iliopsoas-, inguinal-, pubic-, and hip-related groin pains, each with different pathophysiological conditions [5]. Most cases of groin pain syndrome generally improve with appropriate conservative treatment, but there are also cases of prolonged and intractable groin pain [3].

It has been well recognized that the pubic bone serves as the attachment site of several muscles involved in movements of the hip and thigh. The primary muscles that attach to or insert the pubis are adductor longus, adductor brevis, adductor magnus, gracilis, pectineus, rectus abdominis, and external oblique. Magnetic resonance imaging (MRI) of patients with groin pain has revealed that intractable cases present a clear cleft sign near the pubic symphysis (approximately 30–50%), possibly indicative of the pain itself [6,7,8,9]. The cleft sign indicates injury to muscle tendon attachment sites within the pubis and consists of both a superior cleft sign and a secondary cleft sign. The former indicates an injury at the rectus abdominis/adductor longus attachment site, and the latter indicates an injury at the adductor brevis to the margin of the pubic ramus attachment site [10,11]. In a study on cleft signs and MRI findings in athletes, Saito et al. reported that athletes with a positive cleft sign exhibited a significantly longer time to return to play than did other athletes without the cleft sign [8].

The attachment sites of those muscle tendons outlined above are often referred to as “pubic aponeurotic plates” or “pubic plates” [12,13]. In previous anatomical studies on the structure of the pubic aponeurotic plate, different reports suggested that the muscle tendon involved in the cleft sign is attached to the pubis via the pubic ligament or via an enthesis structure that includes cartilage [14,15,16,17]. However, these reports remain controversial, and no consensus has been reached among researchers or studies. Additionally, to the best of our knowledge, no previous study has histologically evaluated what MRI cleft signs represent.

In this study, we aimed to evaluate the structure of the pubic plate histologically using cleft sign-positive and cleft sign-negative cadavers confirmed by MRI and to identify the presence of cartilage in the pubic plate and its location and whether cartilage damage was correlated with the MRI cleft sign. We hypothesized that the pubic plate exhibits a tendon–cartilage–bone enthesis structure at the site of the cleft sign and that the cleft sign indicates a disruption of the cartilage component in the enthesis structure.

## 2. Materials and Methods

**Figure 1:**
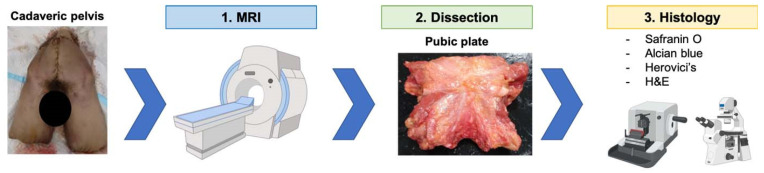
Study design: First, MRI images of freshly frozen human cadaveric pelvises were obtained. Second, the cadavers were dissected to harvest pubic plates. After the decalcification process, pubic plates were cut for histological analysis. H&E: hematoxylin and eosin.

This study was carried out in accordance with the ethical standards of the 1964 Declaration of Helsinki and relevant regulations of the US Health Insurance Portability and Accountability Act (HIPAA). This study was performed at the Steadman Philippon Research Institute and the Steadman Clinic. MRI was performed by an experienced radiologist from Steadman Clinic. Three frozen male human cadaveric pelvises (aged 49, 49, and 35 years) with no evidence of prior injuries or abnormalities were acquired. The outline of this study is shown in Figure 1. The frozen specimens only underwent a single freeze/thaw cycle. MRI and dissection were performed on the same day for each specimen to avoid refreezing.

### 2.1. MRI of Cadavers

After thawing for 24 h, the specimens were imaged using a 3.0T MRI system (Siemens, Erlangen, Germany) as described previously [14]. The specimens were then placed in the supine position, and MRIs were aligned with the cadaveric slice view. The field of view (FOV) was decreased compared to the FOV of a routine pelvic MRI scan. Short T1 inversion recovery (STIR) imaging was performed with the following imaging parameters: repetition time/echo time (TR/TE), 5250/25 milliseconds; 2 mm slice thickness, 1 mm interval; FOV, 245 × 270 mm; matrix size, 512 for coronal sections; TR/TE, 6070/25 milliseconds; 2 mm slice thickness, 1 mm interval; FOV, 245 × 270 mm; and matrix size, 512 for sagittal sections. Two orthopedic surgeons with more than 15 years of clinical experience (S.N. and N.F.) reviewed all MRIs to confirm the presence of the cleft sign. The specimens were identified as cleft sign positive if a hyperintense linear signal from the symphyseal cleft parallel to the inferior margin of the superior pubic ramus or a hyperintense linear signal from the symphyseal cleft parallel to the inferior margin of the inferior pubic ramus were confirmed [11,18].

### 2.2. Cadaver Dissection

**Figure 2:**
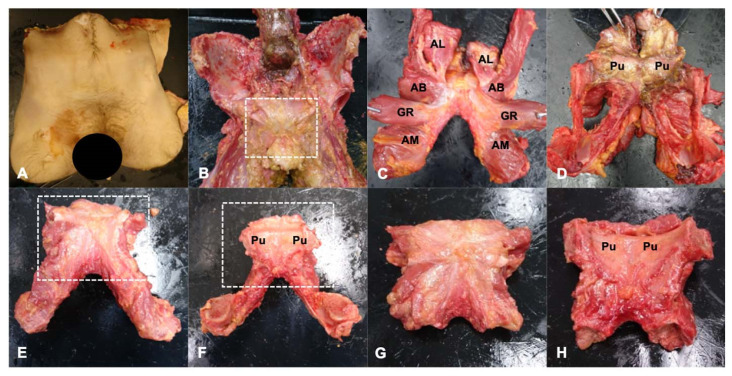
Process of harvesting pubis with pubic plate: (**A**) Bilateral hip cadaver. (**B**) Gross view after removing skin and soft tissue from the muscles attached to the pubic bone. (**C**) Outer side of the separated pubic bone and muscles (the area surrounded by a white dotted box in (**B**). (**D**) Inner side of the separated pubic bone and muscles. (**E**) Outer side of the pubic bone after muscles were removed. (**F**) Inner side of the pubic bone after muscles were removed. (**G**) Outer side of the pubic plate (the area surrounded by a white dotted box in (**E**,**F**). (**H**) Inner side of the pubic plate. AL: adductor longus, AB: adductor brevis, AM: adductor magnus, GR: gracilis, Pu: pubic bone.

After MRI, the cadavers were dissected to harvest the pubic plate for histological analysis. The process of pubic plate harvesting is shown in Figure 2. The specimens were placed in the prone position, and the adductor longus (AL), adductor brevis (AB), adductor magnus (AM), and gracilis (GR) origins were exposed via an open approach. The AL, AB, AM, and GR were followed proximally to their respective origins in the pubic bone. The pubic plate was identified after all muscles were removed, and residual bones were then cut using a bone saw.

### 2.3. Tissue Processing and Segmentation

**Figure 3:**
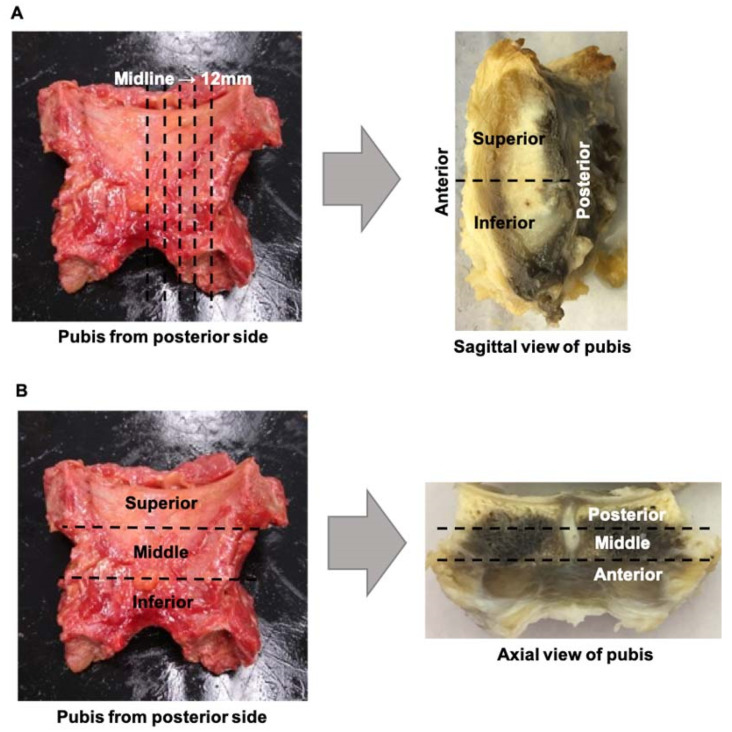
Tissue processing and segmentation: (**A**) For sagittal sectioning, the pubis was sagittally cut at the midline of the pubis first and then 3 mm, 6 mm, 9 mm, and 12 mm from the midline to create serial tissue pieces. Then, each level of the sagittal piece was axially cut into superior and inferior parts. (**B**) For coronal sectioning, the pubis was first axially cut into three pieces: superior, middle, and inferior parts. Then, each part was coronally cut into 3 levels: anterior, middle, and posterior.

Harvested pubic bones were fixed in formalin for 2 weeks and then decalcified in 5% formic acid in water for three months. The cleft sign-negative pubises were cut using different sectioning approaches. One was cut into sagittal sections. The pubis was sagittally cut at the midline (pubic symphysis) first and then in 3 mm, 6 mm, 9 mm, and 12 mm sections to create serial tissue pieces. Due to the need to allow the tissue to fit the cassettes for microtome sectioning, each level of the sagittal piece was then axially cut into superior and inferior parts (Figure 3A). Another cleft sign-negative pubis was cut into coronal sections. Due to the need to allow the tissue to fit the cassettes for microtome sectioning, the pubis was first axially cut into three pieces: superior, middle, and inferior parts. Then, each part was coronally cut into 3 levels: anterior, middle, and posterior. In total, 9 pieces of tissue were cut into coronal sections for histological analysis (Figure 3B). With the cleft sign negative, gross observation was also performed to identify the cartilage structure while making sagittal and coronal pieces.

The cleft sign-positive pubis was cut into sagittal sections in the same way that the cleft sign-negative pubis was cut. All tissue sections were then processed using a tissue processor, and all sections were embedded in paraffin. Afterwards, the samples were cut into 5 µm sections, which were mounted on glass slides. The slides were baked for 3 h at 60 °C for further histological analysis.

### 2.4. Histology

Histological analysis was performed by a single scientist (X.G.) with many years of experience in musculoskeletal research and discussed with other co-authors. Tissue section slides were deparaffinized using xylene and then rehydrated using gradient alcohol to distilled water for further staining. To determine the presence and location of the cartilage, we performed Safranin O and Alcian blue staining using IHC world protocols as previously described [19,20]. Herovici’s staining was also performed as previously described [21,22,23,24]. Hematoxylin and eosin (H&E) staining was performed using a hematoxylin and eosin Y purchased from ANATECH, Inc., in accordance with the manufacturer’s protocol. Microscope images were captured using a Nikon Ti microscope (Nikon, Tokyo, Japan). Large images were first taken automatically at 40× magnification to view the structural tissue components of the public plate using a color camera equipped with a Nikon Ti microscope (Nikon, Tokyo, Japan). Subsequently, 40× individual images were captured at the cartilage area for each staining.

## 3. Results

### 3.1. Magnetic Resonance Imaging

**Figure 4:**
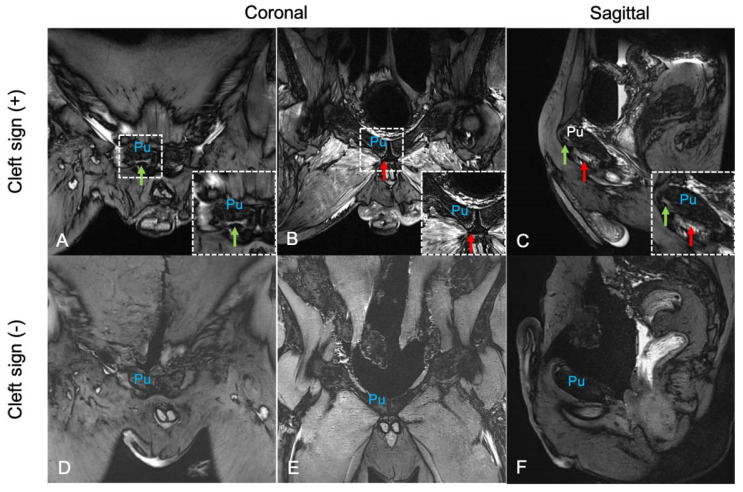
MRI of a cleft sign-positive and cleft sign-negative specimen: (**A**–**C**) MRI of a cleft sign-positive specimen at the coronal and sagittal views. (**D**–**F**) MRI images of a cleft sign-negative specimen at the coronal and sagittal views. The green arrows indicate superior cleft signs, and the red arrows indicate secondary cleft signs. Pu: pubic bone.

Two specimens did not show cleft signs, but one specimen demonstrated superior and secondary cleft signs on both sides of the pubis. Representative MRI coronal and sagittal images of cleft sign-negative and cleft sign-positive specimens are shown in Figure 4.

### 3.2. Gross Observation of the Pubis

**Figure 5:**
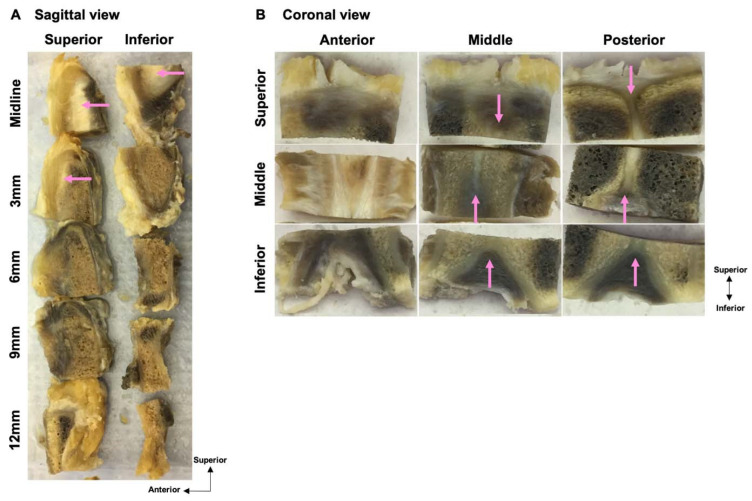
Gross images of sagittal and coronal sections after fixation and decalcification: (**A**) Gross image of sagittal sections. Dense white cartilage was broadly observed at the midline level. Only a small portion of the dense white cartilage was observed in the superior–anterior part of the pubis at the 3 mm sagittal level. (**B**) Gross image of the coronal sections. Cartilage was located at the pubic symphysis and gray area where the muscle transitioned to the tendon. The pink arrows indicate the cartilage and pubic symphysis.

In sagittal images of the pubis (Figure 5A), dense white cartilage was broadly observed within the pubis at the midline level. While only a small portion of the dense white cartilage was observed in the superior–anterior part of the pubis at the 3 mm sagittal level, no dense white cartilage was identified at the 6 mm, 9 mm, or 12 mm sagittal level.

In coronal images of the pubis (Figure 5B) at the superior level, cartilage was located at the center between the trabecular bone (pubic symphysis: pink arrows), where muscle (yellowish part) transitioned to tendon (grayish part) in the middle and posterior parts. At the middle and inferior levels, cartilage was also located at the center between the trabecular bone (pubic symphysis: pink arrow) in the middle and posterior regions. In addition, at the inferior region, the cartilage area appeared gray.

### 3.3. Identification of Cartilage in the Pubic Plate Using Large Microscope Images of Sagittal Sections

**Figure 6:**
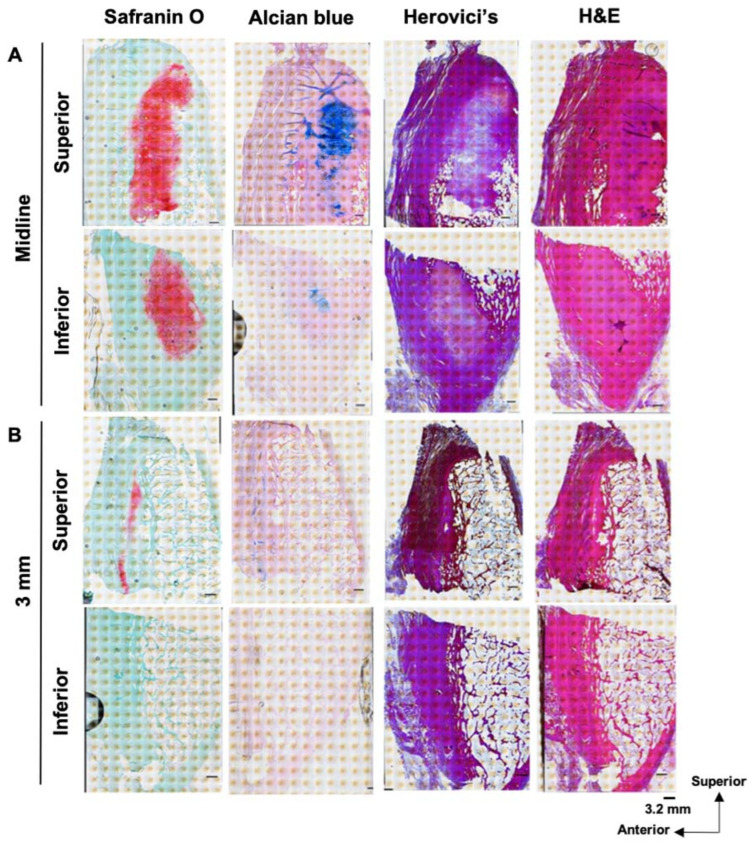
Large composite images of sagittal sections of the pubis without cleft signs: (**A**) Safranin O, Alcian blue, Herovici’s, and H&E staining of the pubis at midline level. The Safranin O-positive matrix was stained orange–red. The Alcian blue-positive matrix was stained blue. Herovici’s staining revealed collagen 1 as a pink–red color and collagen 3 as a dark blue color. H&E staining revealed nuclei in blue and the cytoplasm in red. The cartilage matrix was found to be a redish color. (**B**) Four different stains reveal tissue structures of the pubis at the 3 mm axial level from the midline of a sagittal section. Safranin O staining showed positive orange–red staining in the superior part of the pubic plate, not in the inferior part. Alcian blue also showed weak staining in the middle of the superior part of the pubic plate. Herovici’s staining showed strong pink–red staining in both superior and inferior parts of the pubic plate. H&E staining showed dense red staining in the anterior half of both superior and inferior of the pubic plate. Trabecular bone was found at the posterior of the pubic plate. Scale bars = 3.2 mm (equal to one 40× image). The yellow dots are due to the light shading effect.

**Figure 7:**
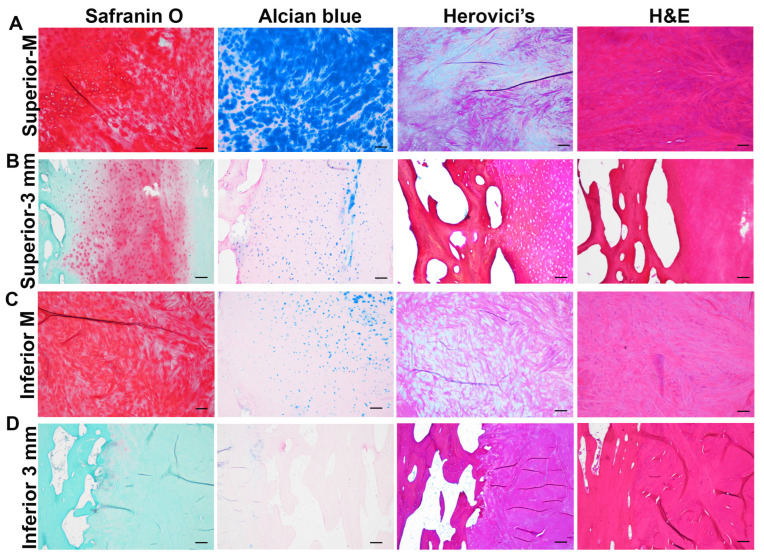
Microscopic verification of the cartilage category with the sagittal sections: (**A**) Superior part of midline. Safranin O staining showed strong orange staining with fibrocartilage morphology. Alcian blue staining also revealed strong blue staining with fibrocartilage-like cells. Herovici’s staining showed light blue staining in the cell body and mixed red and blue staining in the matrix. H&E staining also showed a blue matrix in the cartilage area. (**B**) Superior part at the 3 mm axial level. At this level, only part of the tissue was stained Safranin O-positive between the trabecular bone and tendon tissues for Safranin O, Alcian blue, Herovici’s, and H&E staining. (**C**) Four sections were stained at the inferior part of the midline. Safranin O staining revealed a strongly orange-stained cartilage matrix with fibrocartilage cell morphology. Alcian blue staining revealed a scattered Alcian blue-positive matrix. Herovici’s staining showed a mixed red and blue matrix. H&E staining revealed a blue matrix of fibrocartilage. (**D**) Four sections of the inferior 3 mm axial region. Safranin O staining revealed an orange-colored cartilage matrix. Similar results were observed for the Alcian blue staining. Herovici’s staining revealed mainly a red collagen matrix. H&E staining revealed mainly red staining in the trabecular bone and tendon. Scale bar = 200 µm.

Safranin O staining indicated strongly orange-stained glycosaminoglycan (GAG) in the middle area of the public plate from the superior to the inferior direction (Figure 6A). Notably, there was a relatively weak and small Safranin O-positive matrix on the superior–anterior side (Figure 6A). Alcian blue staining revealed intense blue staining (hyaluronic acid and acid muccin) in the middle area of the superior region, which continued toward the anterior region. However, the stain was localized to a small area within the inferior region (Figure 6A). Herovici’s staining showed dense pink–red collagen 1-positive staining in both the superior and inferior portions of the pubic plate, while the middle part exhibited a mixed blue (collagen 3) and red matrix (collagen 1) color (Figure 6A). H&E staining revealed a bluish area that looked identical to the Safranin O-positive matrix and similar to the cartilage matrix (Figure 6A). At the 3 mm axial level of the sagittal section, a Safranin O-positive matrix was identified on the superior to middle and anterior sides of the pubic plate but not in the inferior half (Figure 6B). In addition, Alcian blue staining revealed weak blue matrix staining in the same area as Safranin O staining (Figure 6B). Herovici’s staining also revealed a mix of blue- and red-colored sections at a location similar to that of the Safranin O-positive region (Figure 6B). Furthermore, H&E staining revealed a similar structure to Herovici’s staining, with the blue matrix occurring in the same location, superior to the middle part of the pubic plate, as Safranin O staining did (Figure 6B). The cartilage is mainly fibrocartilage, as revealed by the morphology of the cartilage cells in Safranin O, Alcian blue, Herovici’s, and H&E staining (Figure 7A–D). Taken together, these results indicate the presence of a cartilage component broadly located at the midline level. However, at the 3 mm axial level, the cartilage component was found to be located in the superior to middle direction on the anterior side. No cartilage was observed at the 6 mm, 9 mm, or 12 mm axial level of the sagittal sections.

### 3.4. Identification of Cartilage in the Pubic Plate Using Large Microscope Images of Coronal Sections

**Figure 8:**
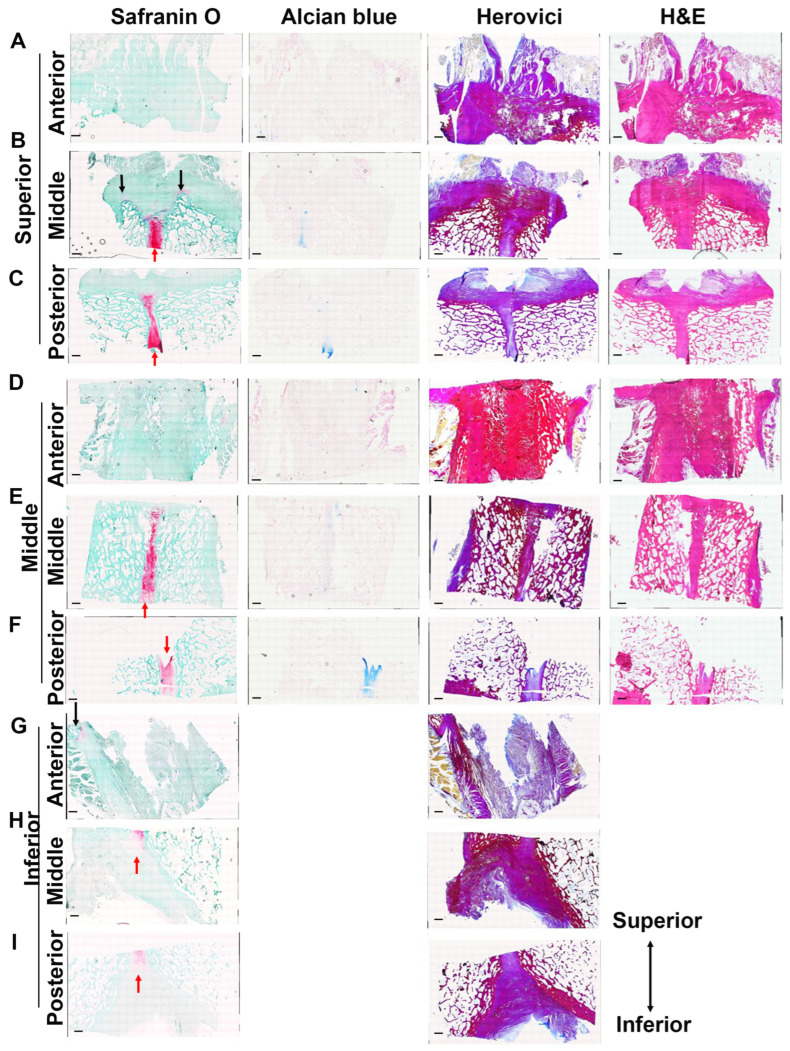
Large composite images of coronal sections of the pubis without cleft signs: (**A**–**C**) Safranin O, Alcian blue, Herovici’s, and H&E staining of the superior part of the coronal section at the anterior, middle, and posterior levels. (**D**–**F**) Safranin O, Alcian blue, Herovici’s, and H&E staining of the middle part of the coronal sections at the anterior, middle, and posterior levels. (**G**–**I**) Safranin O, Alcian blue, Herovici’s, and H&E staining of the inferior part of the coronal sections at the anterior, middle, and posterior levels. Red arrows indicate cartilage at the pubic symphysis. The black arrows indicate cartilage in the muscle–tendon junction. Scale bar = 3.2 mm.

In the superior part of the pubis, Safranin O- and Alcian blue-positive cartilage structures were observed at the center (pubic symphysis) in the middle to posterior parts (Figure 8B,C, red arrows). In addition, a safranin O-positive cartilage structure in the muscle–tendon junction that continued from the pubic symphysis was identified in the middle part (Figure 8B, black arrows). A similar trend was found in the middle and inferior parts (Figure 8E,F,H,I, red arrows), except for the addition of a cartilage structure in the muscle–tendon junction in the inferior–anterior part (Figure 8G, black arrows). All the results indicate that pubic cartilage is located mainly in the pubic symphysis and in the muscle–tendon junction area, which continues from the pubic symphysis at the anterior and middle parts of the pubis.

### 3.5. Cartilage Tears Were Identified in the Cleft Sign-Positive Specimen with Sagittal Sections

**Figure 9:**
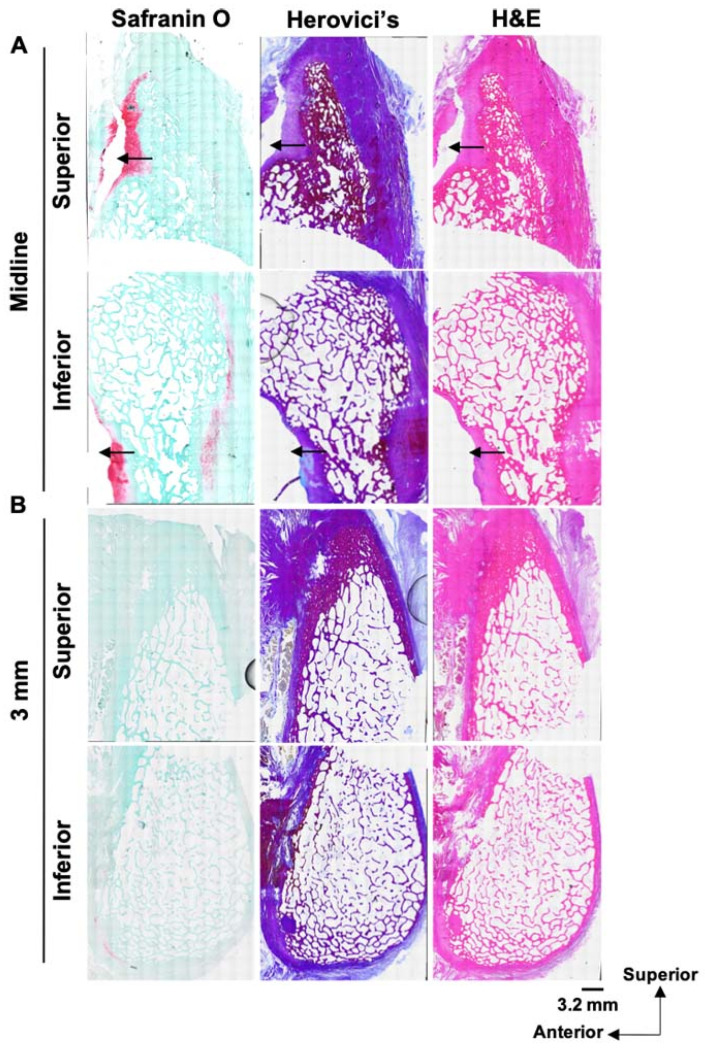
Histological evaluation of sagittal sections of cleft sign-positive pubis: (**A**) Safranin O, Herovici’s, and H&E staining of pubis at the midline. A tear of the Safranin O-positive matrix (orange, red) was observed as indicated by black arrows at the superior and inferior sections. Herovici’s and H&E staining also showed a tear at the same location. (**B**) Safranin O, Herovici’s, and H&E staining 3 mm from the midline at the axial level. A small amount of Safranin O-positive matrix was located on the anterior side of the inferior section. Scale bar = 3.2 mm.

At the midline, we identified cartilage in both the superior and inferior parts, as demonstrated by Safranin O staining (Figure 9A). Further, we identified a cleft on the cartilage in both sections (Figure 9A, black arrows). Hervoci’s and H&E staining also revealed a cleft at the same location (Figure 9A, black arrows). At the 3 mm axial level, a small amount of Safranin O-positive cartilage was located on the anterior side of the inferior section (Figure 9B). At this level, we were not able to identify a cleft (Figure 9B) or much cartilage, possibly because this cadaver possessed a small pubic plate. This result indicates that the cleft sign on MRI represents a disruption of the cartilage component within the pubic symphysis and pubic plate.

## 4. Discussion

The present study confirmed the presence of a fibrocartilage structure in the midline of the pubis (pubic symphysis), which can extend 3 mm laterally at most, using a human pelvic specimen without cleft signs. In the sagittal sections, the cartilage component was broadly located at the midline level. However, the cartilage component was found to be located in the superior to middle direction on the anterior side at the 3 mm axial level. On the basis of the coronal sections, we confirmed that pubic cartilage was located mainly in the pubic symphysis. In addition, more cartilage was observed in the muscle–tendon junction area, which seems to continue from the pubic symphysis. Using a cleft sign-positive cadaver, we further identified a cartilage tear/cleft in the pubic plate that presented as a cleft sign on MRI scans.

Long-standing groin pain is a form of groin pain in which groups of symptoms are reported by the patient for a long period of time, typically more than 12 weeks, and do not respond positively to conservative therapy [25]. Long-standing groin pain is potentially career-ending for elite athletes, and previous studies have shown that athletes with long-standing groin pain exhibit a cleft sign and that such athletes require a significantly longer time to return to play [6,7,8,9]. To investigate the pathogenesis of cleft sign-related long-standing groin pain, we performed several histological evaluations using different staining methods to evaluate the cartilage, bones, and tendons. Our results indicate the presence of a cartilage structure in the pubic plate composing the enthesis structure of the muscle tendon–cartilage/bone–bone. De Maeseneer et al. performed imaging, anatomical, and histological evaluation of the abdominal muscles and adductor tendon insertions; the authors reported that the AL tendon fibers were inserted perpendicularly into the bone through a fibrocartilage enthesis and cross-connected along the anterior pubic ligament into the contralateral tendon [14]. Although they found that fibrocartilage entheses existed at the AL tendon insertion site, their histological evaluation was not comprehensive enough because they performed only H&E staining of coronal sections. In our study, we confirmed the presence of cartilage through H&E staining as well as Safranin O, Alcian blue, and Herovici’s staining. Two cartilage-specific staining methods, Safranin O staining (for GAG) and Alcian blue staining (for hyaluronic acid and aciduccin), revealed the cartilage to be fibrocartilage and that the location was mainly between the midline and 3 mm in the sagittal view. Herovici’s staining also revealed collagens 1 and 3 in the transition area from bone to cartilage.

**Figure 10:**
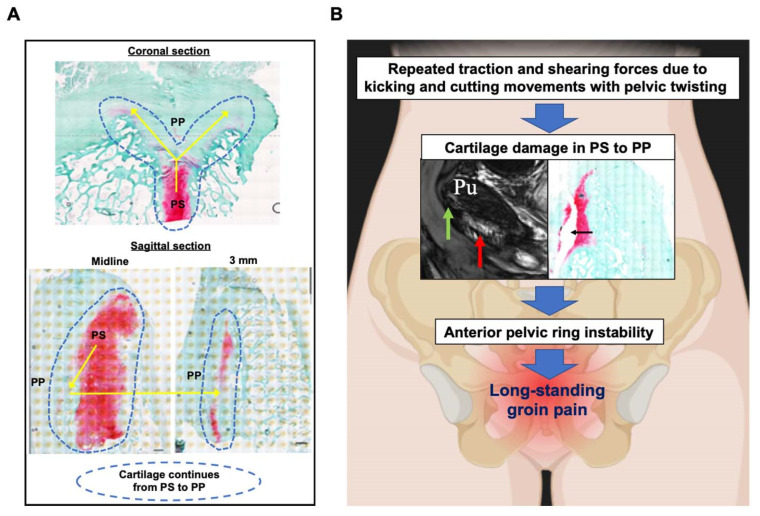
Summary of the main findings and suggested pathogenesis of long-standing groin pain. (**A**) Histological analysis of pubic plates in this study suggested that the cartilage component of the pubic plate extends from the pubic symphysis. (**B**) Repeated traction and shearing forces due to kicking and cutting movements with pelvic twisting cause damage to the cartilage in the pubic symphysis to the pubic plate, resulting in long-standing groin pain. PS: pubic symphysis, PP: pubic plate.

Notably, in the coronal section of a cleft sign-negative cadaver, the cartilage component continued from the pubic symphysis all the way to the enthesis structure in the pubic plate. According to the sagittal section of a cleft sign-negative cadaver, the cartilage component also seems to extend from the pubic symphysis to the enthesis structure in the pubic plate. According to previous studies that originally defined the cleft sign, the fissure within the pubic symphysis was originally called the central cleft [10]. The contrast agent is injected into the pubic symphysis, after which the agent leaks into the inferior pubic branch, termed the “secondary cleft sign” [10]. Subsequently, leakage of contrast agent into the superior pubic branch was also identified after the contrast agent was injected into the pubic symphysis, referred to as the “superior cleft sign” [11]. Given these definitions, we believe that the cartilage component within the pubic plate related to the cleft sign actually continues from the pubic symphysis (Figure 10A) and that the cleft sign represents cartilage damage continuing from the pubic symphysis to the pubic plate.

Furthermore, we found that a cartilage tear correlated with an MRI image of the cleft in the pubic plate and could be related to groin pain. Since the Doha Agreement was established, previous studies have reported that two-thirds of groin pain was adductor muscle-related and pubic bone-related groin pain was only 4% [26,27]. However, the Doha Agreement is based on physical findings alone. Without the use of diagnostic imaging, it is difficult to distinguish between adductor muscle-related and pubic bone-related pain [28]. Our study revealed that the cleft sign, which is considered a pathogenesis marker of long-standing groin pain, is indicative of cartilage damage continuing from the pubic symphysis to the pubic plate. It is possible that this pathology may underlie long-standing groin pain, regardless of whether it is adductor-related or pubic-related. Therefore, it is important to confirm the presence of cleft signs by MRI, including the pelvic region, even in patients classified as adductor-related by the Doha Agreement to rule out long-standing groin pain.

To date, the mechanism of long-standing groin pain is not well understood. It has been reported that repetitive and quick accelerating or decelerating movements, including abrupt changes in direction and kicking and cutting movements, can be specific physical inducers of such pain [29]. Such acute trauma may lead to an overwhelming amount of mechanical stress on the pubic symphysis, as well as the pubic plate, and ultimately disrupt the cartilage structure within those areas. In a study of 66 male athletes with groin pain, Hall et al. reported that 55% of the athletes found to be cleft sign positive additionally had anterior pelvic ring instability [30]. Chronic anterior pelvic instability is a pathological movement of the pubic symphysis associated with axial loading. As it is not a common condition, diagnosis is often delayed, and disability is increased in pathogenic individuals [31]. On the basis of the results of our study, we speculate that cartilage damage continuing from the pubic symphysis to the pubic plate may lead to anterior pelvic ring instability and long-standing groin pain. Our proposed mechanism of long-standing groin pain in patients tested as cleft sign positive and often recognized as adductor-related groin pain is shown in Figure 10B.

This study has several limitations. First, this study included only one cleft sign-positive cadaver. Therefore, a follow-up study using more cadavers positive for cleft signs is necessary to explore cartilage damage in the pubic plate with different sectioning sequences (coronal section, etc.) for histological evaluation. Second, none of the radiologists evaluated the MRI images, and the health status of the patient was unknown at the time of death. Third, although there was no history of injury to the cadaver of a human patient, we detected the cleft sign via MRI. Since the MRI findings and histological analysis results were consistent, we believe that the cleft sign found on the MR images represents similar cartilage damage in the pubic plate at the level of the cartilage structure in human patients.

Of note, the high-resolution radiological techniques are continuously expanding. Pušnik et al. performed the cadaveric study to compare three-dimensional (3D) reconstructions of median and ulnar nerves acquired with tomographic high-resolution ultrasound (HRUS) and magnetic resonance microscopy (MRM) and assess their capacity to depict intraneural anatomy [32]. They concluded that MRM demonstrated a more detailed fascicular depiction compared to 3D HRUS, with a greater capacity for visualizing smaller fascicles. Although there have been no previous reports of the application of high-resolution radiological techniques in the imaging diagnosis of cleft signs, the application of their high-resolution radiological techniques to cleft signs may lead to a more accurate pathophysiological evaluation of cleft signs.

## 5. Conclusions

This study elucidated the existence of a cartilage component extending from the pubic symphysis to the pubic plate. Discern of the cleft sign signifies a disruption in the cartilage component within this specific area. The observed cartilage damage in these regions may represent a pathogenic change intricately linked to long-standing groin pain.

## Data Availability

The original contributions presented in this study are included in the article; further inquiries can be directed to the corresponding author.

## Abbreviations

MRI: magnetic resonance imaging, FOV: field of view, STIR: short T1 inversion recovery, TR/TE: repetition time/echo time, AL: adductor longus, AB: adductor brevis, AM: adductor magnus, GR: gracilis, H&E: hematoxylin and eosin, GAG: glycosaminoglycan.
